# LINC00511/miRNA-143-3p Modulates Apoptosis and Malignant Phenotype of Bladder Carcinoma Cells via PCMT1

**DOI:** 10.3389/fcell.2021.650999

**Published:** 2021-04-09

**Authors:** Li-Ming Dong, Xi-Ling Zhang, Ming-Huan Mao, Yan-Pei Li, Xi-Yan Zhang, Dong-Wei Xue, Yi-Li Liu

**Affiliations:** ^1^Department of Urologic Surgery, The Fourth Affiliated Hospital of China Medical University, Shenyang, China; ^2^Department of General Surgery, The Fourth Affiliated Hospital of China Medical University, Shenyang, China

**Keywords:** bladder cancer, apoptosis, PCMT1, LINC00511, invasion

## Abstract

Bladder cancer has easy recurrence characteristics, but its occurrence and development mechanism are still unclear. Non-coding RNA is a kind of RNA that exists widely and cannot be translated into proteins, which has played a key role in the regulation of biological functions of tumor cells. However, the regulation mechanism of non-coding RNA on bladder tumors is not fully understood. By microarray analysis and database analysis, we found that LINC00511 was significantly highly expressed in bladder cancer. The expressions of LINC00511, miR-143-3p, and PCMT in bladder cancer tissues and cells were detected by quantitative reverse transcription–polymerase chain reaction. The relationship between the expressions of miR-143-3p and PCMT1 and the clinicopathological parameters of the tumor was analyzed. The proliferation and invasion of bladder cancer cells were detected by MTT assay and Transwell assay. The expression levels of E-cadherin and vimentin in bladder cancer cells were detected by Western blot. Cell apoptosis was detected by flow cytometry. *In vivo*, TCCSUP or SW780 cells were inoculated into BALB/c nude mice to detect tumor volume and weight. Bioinformatics and dual luciferase reporter gene were used to analyze the relationship between LINC00511 and miR-143-3p and its downstream target gene PCMT1. The results showed that LINC00511 could target miR-143-3p/PCMT1 to regulate the proliferation, migration, and apoptosis of bladder cancer TCCSUP or SW780 cells and promote the occurrence and development of bladder cancer.

## Introduction

Bladder cancer is a kind of urogenital tumor with high morbidity and mortality (Chen et al., 2020; Woła̧cewicz et al., 2020). There are two types of bladder cancer: non-muscular invasive bladder cancer (TA and T1) and muscular invasive bladder cancer (T2–4) (Kunze et al., 1983). Although radical surgery and radiotherapy are effective, one-quarter of patients with muscle-invasive bladder cancer still have a poor prognosis (Sternberg et al., 2015). In addition, approximately 75% of patients presented with non-muscular invasive bladder cancer, and the other 25% presented with muscular invasive bladder cancer or metastatic tumor (Burger et al., 2013). Patients with non-muscular invasive bladder cancer have up to 50% risk of recurrence and 20% risk of progression within 5 years. For high-risk patients, cystoscopy and urinalysis every 3 months are required routinely (Cambier et al., 2016; Chang et al., 2016). Although cystoscopy is an effective method for detecting bladder cancer, it can also produce several adverse reactions. Common adverse reactions include infection, dysuria, frequent urination, and hematuria. Therefore, it is very important to study the pathogenesis of bladder cancer and control the recurrence of bladder cancer. It is indispensable to develop non-invasive diagnostic tools for the screening and monitoring of bladder cancer to improve the quality of life of patients.

Non-coding RNA refers to a kind of RNA that cannot be translated into proteins in cells; however, it can regulate various biological functions of cells (Yao et al., 2019; Statello et al., 2020). As a signal molecule, bait molecule, skeleton molecule, and guide molecule, it can directly participate in the epigenetic inheritance and transcriptional and posttranscriptional regulation of cells and genes and also play a key role in the occurrence and development of tumors. More and more studies have revealed that lncRNA can interact with DNA, RNA, and proteins to participate in histone modification, chromatin remodeling, and other cell biological processes (Fabbri et al., 2019). Endogenous competitive RNA (ceRNA) is one of the mechanisms by which lncRNAs perform biological functions, refer to that lncRNAs act as “sponge” to specifically bind miRNA, inhibit the function of miRNA, indirectly regulate protein-coding genes, and thus affect the biological function of cells (Schmitz et al., 2016). Clinically, there are many types of lncRNA associated with bladder cancer, including HOTAIR, MALAT1, H19, etc. (Sun et al., 2015; Lv et al., 2017; Qi et al., 2019), all of which play an important role in the occurrence and development of bladder cancer. Strengthening the understanding of the role of non-coding RNA in bladder cancer can improve the early diagnosis and treatment of laryngeal cancer and hopefully improve the quality of life after surgery.

In this study, lncRNAs differentially expressed in bladder cancer were investigated by chip technology. The high expression of LINC00511 in bladder cancer was determined by computer database analysis and collection of bladder cancer tumor samples and adjacent tissues. The purpose of this study was to investigate the relationship between LINC00511 on proliferation, invasion, and apoptosis of bladder cancer cells and to explore its regulatory pathways.

## Materials and Methods

### Tissue Specimen

Tissue specimens were selected from the specimen bank of The Institute of Urology, China Medical University, with complete clinical data and definite diagnosis of bladder cancer (47 cases of cancer tissue and 47 cases of adjacent tissue). The experiment involving human tissue was authorized and approved by the ethics committee of China Medical University. The patients provided their written informed consent to participate in this study.

### Gene Expression Microarray Analysis

The array images were analyzed using Agilent Feature Extraction software (version 11.0.1.1). Subsequently, quantile normalization and data processing were performed by the GeneSpring GX v12.1 software package (Agilent Technologies, CA, United States). LncRNAs that have flags present or marginal in at least 50% of samples were used for data analysis. To identify differentially expressed lncRNAs, Welch *t* test was performed on the normalized intensity of each lncRNA, followed by Benjamini and Hochberg method to adjust *p* values. Differentially expressed lncRNAs were defined as lncRNAs with absolute fold change ≥1.5 and adjusted *p* < 0.05.

### Animals

BALB/c nude mice (female; 4–6 weeks, Dashuo Experimental Animal Center, Chengdu, China) were used for the experiment of Xenograft; TCCSUP and SW780 cells (5 × 10^6^) were injected subcutaneously. Tumor volume and weight were recorded once a week. The animal study was reviewed and approved by China Medical University.

### Cell Culture

All bladder cancer cell lines were purchased from Cell Resource Center (Chinese Academy of Sciences, Shanghai, China) and cultured in a medium containing 10% fetal bovine serum (McCoy 5A) and 1640 (Hyclone) and in a constant temperature environment containing 5% CO_2_ and 37∘C.

The TCCSUP and SW780 cell lines stably transfected with sh-LINC00511/sh-NC were constructed by Wuhan Biofavor Biotech Service Co., Ltd. (Wuhan, China).

### Colony Formation

TCCSUP and SW780 cells in the logarithmic growth phase were prepared and cultured in dishes containing culture medium for 2–3 weeks. The culture was terminated when visible clones appeared in the Petri dish, stained with Giemsa staining solution, and the number of clones was calculated.

### Quantitative Reverse Transcription–Polymerase Chain Reaction

Total RNA samples were extracted using TRIzol (Invitrogen, Carlsbad, CA, United States). Real-time reverse transcription–polymerase chain reaction (RT-PCR) was performed in 7500 Fast Real-Time PCR System (Applied Biosystems) using SYBR^®^ Green Master Mix (Thermo Fisher Scientific, Inc., MA, United States). Comparative threshold cycle (*C*_*t*_) method was used to calculate the level of mRNAs and miRNAs.

### RNA Immunoprecipitation Assay

The combination of LINC00511 and Ago2 was detected by the RNA immunoprecipitation (RIP) kit (Millipore, Bedford, MA, United States). All experiments were carried out according to kit instructions. The antibody was anti-Ago2 (ab57113, Abcam, MA, United States). Anti–immunoglobulin G (Santa Cruz Biotechnology, TX, United States) was used as the control group.

### RNA Pull-Down

TCCSUP cells were transfected with 50 nM biotin-labeled Wt-bio-miR-143-3p and Mut-bio-miR-143-3p (Sangon Biotech, Shanghai, China). The experimental method was described previously (Zhao et al., 2019). The binding RNA was purified with Trizol, and the expression of LINC00511 was determined.

### Transfection

The TCCSUP and SW780 cells were transfected until the cell density reached 80% confluency. The plasmid of LINC00511 and PCMT1/sh-PCMT was constructed by Genechem (Shanghai, China). miR-143-3p mimics and inhibitor were constructed by Ribo (Ribo Bio, Guangzhou, China). The plasmids were transfected by electroporation.

### Western Blot

Western blot was performed as described previously (Li et al., 2020). The primary antibody of PCMT1 (1:500), E-cadherin (1:500), vimentin (1:1,000), and GAPDH (1:2,000) were purchased from ProteinTech (Wuhan, China).

### MTT Assay

TCCSUP and SW780 were plated in 96-well plates. MTT solution (0.5 mg/mL) was purchased from Beyotime Biotechnology (Shanghai, China) and incubated for 4 h at 37°C. Then 150 μL dimethyl sulfoxide incubated for 15 min. The absorbance was measured by spectrophotometer (NanoDrop Technologies, DE, United States) at 493 nm.

### Fluorescence *in situ* Hybridization Assay

The probes of LINC00511, 18s RNA, and U6 RNA were synthesized; Ribo fluorescence was used for fluorescence *in situ* hybridization (FISH) kit (Ribo Bio, Guangzhou, China). Nucleus was stained with DAPI. The experimental steps were performed as described (Guo et al., 2018).

### Cell Apoptosis Assay

Cells were treated with serum starvation. Apoptosis was analyzed using an apoptosis detection kit (Invitrogen, MA, United States) according to the instructions. Apoptosis rate was calculated by the proportion of early apoptotic cells and late apoptotic cells in the total number of cells.

### Luciferase

The 3′-UTR of LINC00511/PCMT1 including the wild type (WT) binding sites or mutant (MUT) was inserted into pmirGLO vector. The LINC00511/PCMT1 reporter plasmids were cotransfected with miR-143-3p mimics or mimics-NC. Luciferase activities were tested using a luciferase reporter assay kit (Promega, WI, United States).

### Matrigel Transwell Assay

Twenty-four-well Matrigel Transwell (Corning, NY, United States) was used to investigate cell invasion; 2 × 10^5^ TCCSUP or SW780 was seeded on precoated Matrigel (1 μg/μL, BD Biosciences, CA, United States). Medium including fetal bovine serum was used to stimulate invasion in the bottom of wells. After 48 h, the invasion cells were stained by 0.1% crystal violet solution.

### *In situ* Hybridization

*In situ* hybridization (ISH) was performed as previously described (Yang et al., 2013). The digoxigenin-labeled LINC00511-specific probe was 5′-TTCTC AAAGC GAGTC GACGA ACCCA CACCC TGACT TACAC-3′.

### Statistical Analysis

Data are shown as mean ± SD. Student *t* test or one-way analysis of variance was used to compare the groups. *p* < 0.05 was considered significant.

## Results

### LINC00511 Was Highly Expressed in Bladder Cancer

**FIGURE 1:**
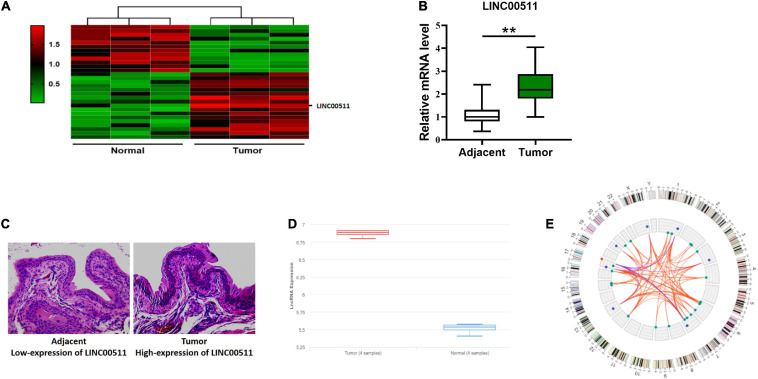
High expression of LINC00511 in bladder tissues. **(A)** Microarray analysis of the differentially expressed lncRNAs between bladder samples and non-tumor samples. **(B)** LINC00511 level in fresh bladder samples and adjacent tissues was measured using quantitative RT-PCR. *n* = 47; **p < 0.01. **(C)** Representative images of LINC00511 expression in bladder tissues analyzed using ISH. **(D)** The expression of LINC00511 bladder samples and non-tumor samples predicted by lnCAR (https://lncar.renlab.org/). **(E)** LINC00511 correlation mRNA network diagram predicted by lnCAR.

We detected differentially expressed lncRNAs in clinical samples by microarray and found that LINC00511 expression was increased in bladder cancer (Figure 1A). Similarly, the clinical samples collected through PCR detection found that LINC00511 was elevated in the cancer tissues compared to the adjacent tissues (Figure 1B). *In situ* hybridization also showed that LINC00511 was more positive in bladder cancer tissues (Figure 1C). According to the lnCAR database, LINC00511 was highly expressed in bladder cancer (Figure 1D). The relative ceRNA network in Figure 1E indicates that LINC00511 may play a biological role by interacting with microRNAs. The above results suggest that LINC00511 may be involved in the development of bladder cancer.

### Silencing LINC00511 Alleviated the Malignancy of Bladder Cancer Cells

**FIGURE 2:**
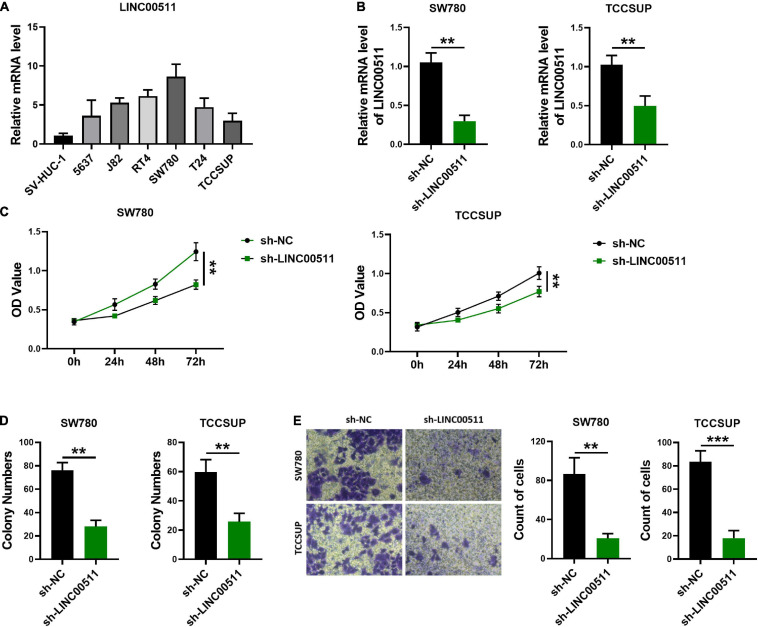
Knockdown of LINC00511 attenuated bladder cancer cells proliferation, colony formation, and invasion. **(A)** The level of LINC00511 was determined using quantitative RT-PCR in six human bladder cancer cell lines, including 5637, J82, RT4, SW780, T24, and TCCSUP, compared to SV-HUC-1, the human bladder cell biochemistry Pillon, *n* = 4. **(B)** Detection of LINC00511 expression in TCCSUP and SW780 stably transfected cell lines. *n* = 6; **p < 0.01. **(C)** MTT assays were performed to assess the proliferation of the TCCSUP and SW780 in response to knockdown LINC00511. *n* = 10; **p < 0.01. **(D)** The colony formation capacity of the TCCSUP and SW780 after silencing LINC00511. *n* = 4; **p < 0.01. **(E)** Transwell invasion assay of TCCSUP and SW780 cells after silencing LINC00511. *n* = 4; **p < 0.01; ***p < 0.001.

To determine the role of LINC00511 in bladder cancer, we first screened the expression levels of LINC00511 in various bladder cancer cell lines (Figure 2A) and selected SW780 with a relatively high expression of LINC00511 and TCCSUP with relatively low level of LINC00511 for subsequent experiments. Lentivirus infection was used to construct LINC00511 knockdown stable cell lines (Figure 2B). We found that silencing LINC00511 inhibited the proliferation (Figure 2C), clone formation (Figure 2D), and invasion (Figure 2E) of TCCSUP and SW780.

### Down-Regulation of LINC00511 Expression Inhibited Tumor Growth *in vivo*

**FIGURE 3:**
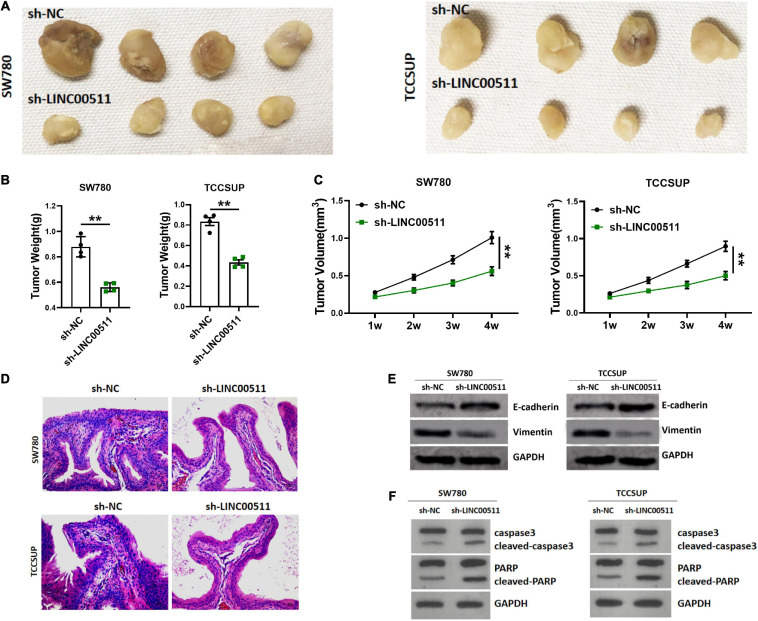
Silencing of LINC00511 inhibited bladder tumor growth *in vivo*. **(A)** Representative images of tumors injected with TCCSUP and SW780 cells, *n* = 4. **(B)** The weight of tumors. *n* = 4; **p < 0.01. **(C)** The tumor volumes. *n* = 4; **p < 0.01. **(D)** Representative ISH image showing LINC00511 expression in xenograft tumors. **(E)** E-cadherin and vimentin proteins levels in xenograft tissues were measured by Western blot. *n* = 4. **(F)** Apoptosis of xenograft was measured by Western blot, *n* = 3.

The experiment of the xenograft tumor model in nude mice showed that stable silencing TCCSUP and SW780 cells were injected subcutaneously into the right flank (Figure 3A). The results showed that silencing LINC00511 could reduce the weight (Figure 3B) and volume of xenograft tumors (Figure 3C). Based on ISH results, the silence of LINC00511 is verified (Figure 3D). Furthermore, LINC00511 knockdown significantly increased the epithelial marker E-cadherin and decreased the mesenchymal marker vimentin (Figure 3E). And then, the expressions of apoptosis-related proteins caspase 3 and PARP in xenograft tumors were detected. We found that cell apoptosis increased after LINC00511 knockdown, which may be one of the mechanisms of sh-LINC00511 inhibiting tumor growth (Figure 3F).

### LINC00511 Competitively Bound With miR-143-3p

**FIGURE 4:**
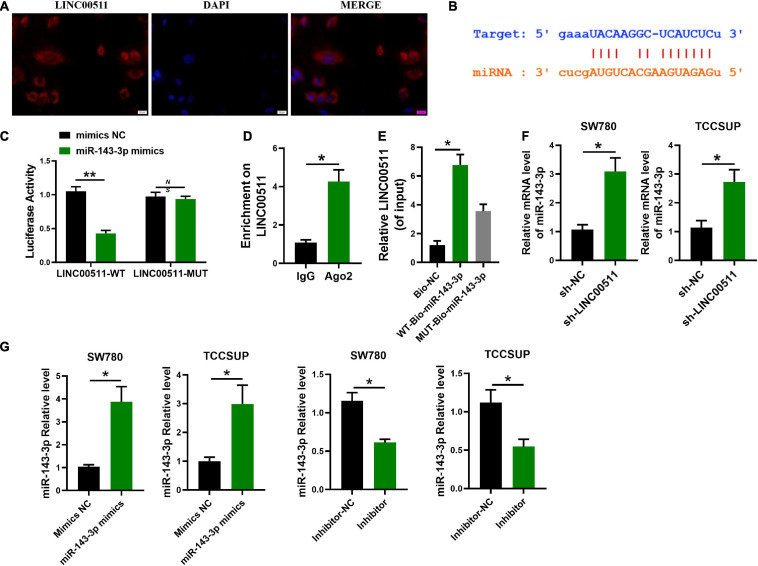
miR-143-3p is a target of LINC00511. **(A)** LINC00511 was mainly expressed in the cytoplasm of TCCSUP cells. *n* = 3. **(B)** The binding sites for LINC00511 in the miR-143-3p sequence were predicted by biological prediction website. **(C)** TCCSUP cotransfected with miRNAs (mimics NC or miR-143-3p mimics) and a reporter vector containing LINC00511 segments (WT or MUT) that bind to miR-143-3p. *n* = 4; **p < 0.01. **(D)** RIP detection of LINC00511 binding to AGO2, AGO2: argonaute 2; *n* = 3; *p < 0.05. **(E)** RNA pull-down analysis of the binding of LINC00511 to miR-143-3p. *n* = 3; *p < 0.05. **(F)** The regulation of miR-143-3p expression by LINC00511 was detected by quantitative RT-PCR (qRT-PCR). *n* = 6; *p < 0.05. **(G)** The efficiency of miR-143-3p mimics and inhibitor was detected by qRT-PCR. *n* = 3; *p < 0.05. WT, wild type; MUT, mutant.

In order to delve the potential mechanism of LINC00511, we first explored its localization. FISH showed that LINC00511 mainly localized in the cytoplasm (Figure 4A). It suggests that LINC00511 may play a role by endogenous competing with microRNA. LINC00511 was predicted to potentially bind to miR-143-3p using the lncRNASNP2 and miRanda databases^1^^2^, respectively. The binding site in miR-143-3p and LINC00511 was identified (Figure 4B). In addition, the luciferase assay showed that miR-143-3p down-regulated the luciferase activity of the LINC00511–WT vector (Figure 4C). RIP/RNA pull-down was used to further verify the interrelationship between LINC00511 and miR-143-3p (Figures 4D,E). Moreover, the level of miR-143-3p increased after the knockdown of LINC00511 (Figure 4F).

### LINC00511 Functioned in TCCSUP and SW780 Proliferation and Invasion by Regulating miR-143-3p

**FIGURE 5:**
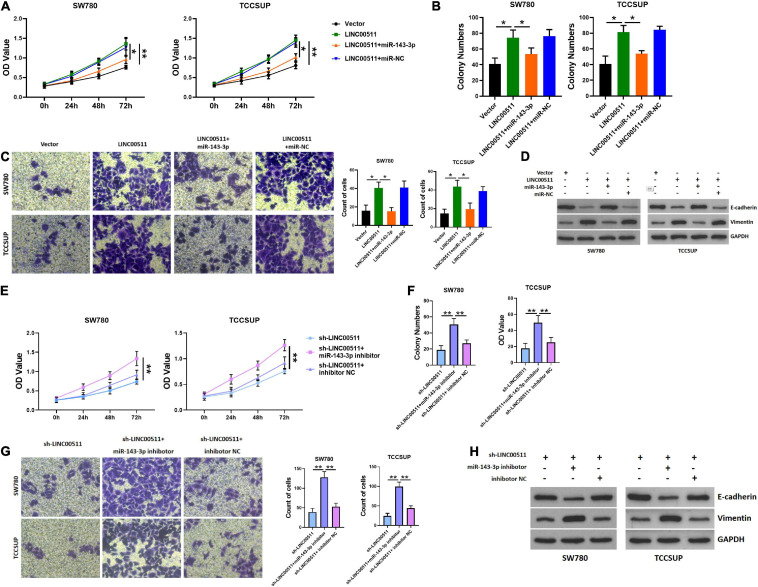
LINC00511 negatively regulated miR-143-3p involved in bladder cancer cell proliferation and invasion. **(A)** MTT assays were used to test the proliferation of TCCSUP and SW780 in response to forced expression LINC00511 or miR-140-5p mimics. *n* = 10; *p < 0.05; **p < 0.01. **(B,C)** The colony formation and Transwell invasion assay of TCCSUP and SW780 cells transfected with LINC00511 plasmid or transfected with miR-143-3p mimics simultaneously. *n* = 4; *p < 0.05. **(D)** E-cadherin and vimentin proteins were determined using Western blot after transfected with LINC00511 plasmid or miR-143-3p mimics. *n* = 3. **(E)** MTT assays were used to detect the proliferation of TCCSUP and SW780 in response to transfect with miR-143-3p inhibitor in LINC00511 knockdown cells. *n* = 10; *p < 0.05; **p < 0.01. **(F,G)** The colony formation and Transwell invasion assay of TCCSUP and SW780 cells transfected with miR-143-3p inhibitor after silencing LINC00511. *n* = 4; *p < 0.05. **(H)** E-cadherin and vimentin proteins were determined using Western blot after being transfected with miR-143-3p inhibitor in LINC00511 knockdown cells. *n* = 3.

We further tested whether LINC00511 functions through miR-143-3p. Initially, the efficiency of miR-143-3p mimic and inhibitor was verified in Figure 4G. Additionally, miR-143-3p mimics inhibited the proliferation and invasion of the TCCSUP and SW780 cells induced by LINC00511 (Figures 5A–C). Similarly, the epithelial–mesenchymal transition (EMT) effect of LINC00511 was reversed by the miR-143-3p mimics (Figure 5D). On the contrary, the sh-LINC00511 inhibition on proliferation, invasion, and EMT of TCCSUP and SW780 were reversed by miR-143-3p inhibitor (Figures 5E–H). Thus, LINC00511 functions as an oncogene in bladder cancer by regulating miR-143-3p.

### LINC00511 Regulated Bladder Cancer Cell Apoptosis Through miR-143-3p

**FIGURE 6:**
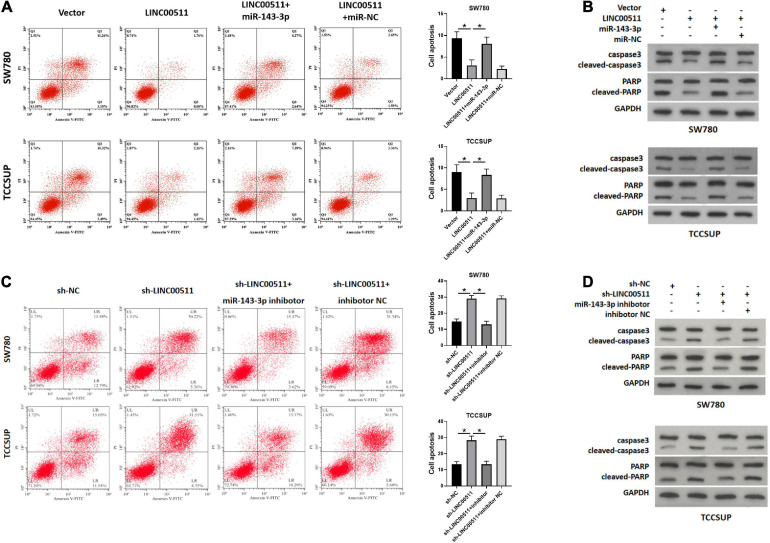
LINC00511 negatively regulated miR-143-3p involved in bladder cancer cell apoptosis. **(A,B)** Apoptosis of TCCSUP and SW780 cells in response to forced expression LINC00511 or miR-143-5p mimics was detected by flow cytometry and Western blot. *n* = 3; *p < 0.05. **(C,D)** Apoptosis of TCCSUP and SW780 cells transfected with miR-143-5p inhibitor after silencing LINC00511 was detected by Flow cytometry and Western blot. *n* = 3; *p < 0.05.

As displayed in Figure 6A, the apoptosis rate was decreased after overexpression LINC00511, but was restored after treatment with miR-143-3p mimics. Conversely, knockdown of LINC00511 resulted in apoptosis of TCCSUP and SW780, and miR-143-3p mitigated this effect (Figure 6C). The results for apoptosis-related proteins showed the same results (Figures 6B,D).

### LINC00511 Modulated the Expression of the miR-143-3p Target PCMT1

**FIGURE 7:**
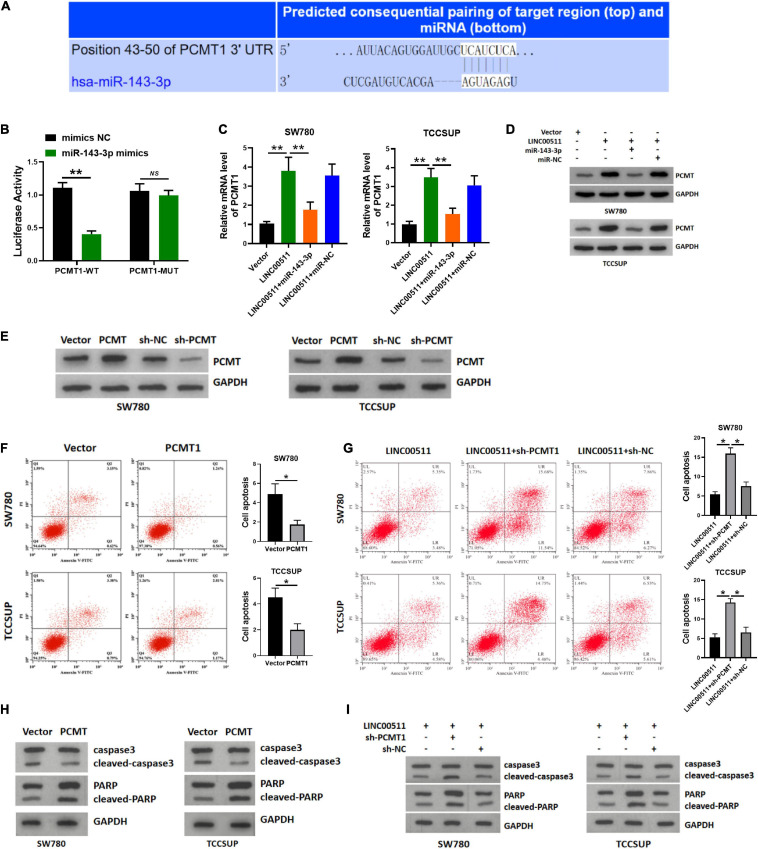
LINC00511 modulated the expression of PCMT1 by targeting miR-143-3p. **(A)** The predicted binding site between PCMT1 and miR-143-3p. **(B)** Luciferase activity was determined cotransfected with miR-143-3p and reporter vector containing PCMT1 WT or MUT 3’-UTR segments that bind to miR-143-3p. *n* = 4; **p < 0.01. **(C)** PCMT1 expression was tested in TCCSUP and SW780 cells using quantitative RT-PCR after the transfection of LINC00511 plasmid or miR-143-3p mimics. *n* = 6; **p < 0.01. **(D)** PCMT1 protein level was tested in TCCSUP and SW780 cells by Western blot after transfection of LINC00511 plasmid or miR-143-3p mimics. *n* = 3. **(E)** The efficiency of PCMT plasmid and sh-RNA. *n* = 3. **(F,H)** Apoptosis of TCCSUP and SW780 cells transfected with PCMT1 detected by flow cytometry and Western blot. *n* = 3; *p < 0.05. **(G,I)** Knocking down PCMT partially reduced the inhibitory effect of LINC00511 on apoptosis of TCCSUP and SW780 cells detected by flow cytometry and Western blot. *n* = 3; *p < 0.05.

Using TARGETSCAN7.2 software^3^, we found that miR-143-3p directly bound to L-isoaspartate (D-aspartate) *O*-methyltransferase PCMT1, a protein in human cancer was generally cognized (Figure 7A). The miR-143-3p mimics down-regulated the luciferase activity of the WT PCMT 3′-UTR in the luciferase assay (Figure 7B). Next, we explored whether LINC00511 modulates the expression of PCMT1 by targeting miR-143-3p. The PCMT1 mRNA and protein levels were increased in LINC00511 overexpression TCCSUP and SW780 cells and were decreased in cells transfected with the miR-143-3p mimics (Figures 7C,D). Based on these results, LINC00511 negatively modulates miR-143-3p expression and targets PCMT1. Our previous studies have shown that PCMT1 is an adverse prognostic biomarker involved in bladder cancer cell migration and invasion by regulating EMT-related genes (Dong et al., 2018). However, the effect of PCMT1 on apoptosis remains unclear. In order to verify the effect of PCMT on apoptosis, we first confirmed the efficiency of PCMT plasmid and sh-RNA (Figure 7E). Here, our results demonstrated that PCMT1 inhibited bladder cancer cell apoptosis, and silencing PCMT reduced the inhibitory effect of LINC00511 on apoptosis (Figures 7F–I).

### Correlations Between the LINC00511 and miR-143-3p and PCMT1 Levels in Human Bladder Tissues

**FIGURE 8:**
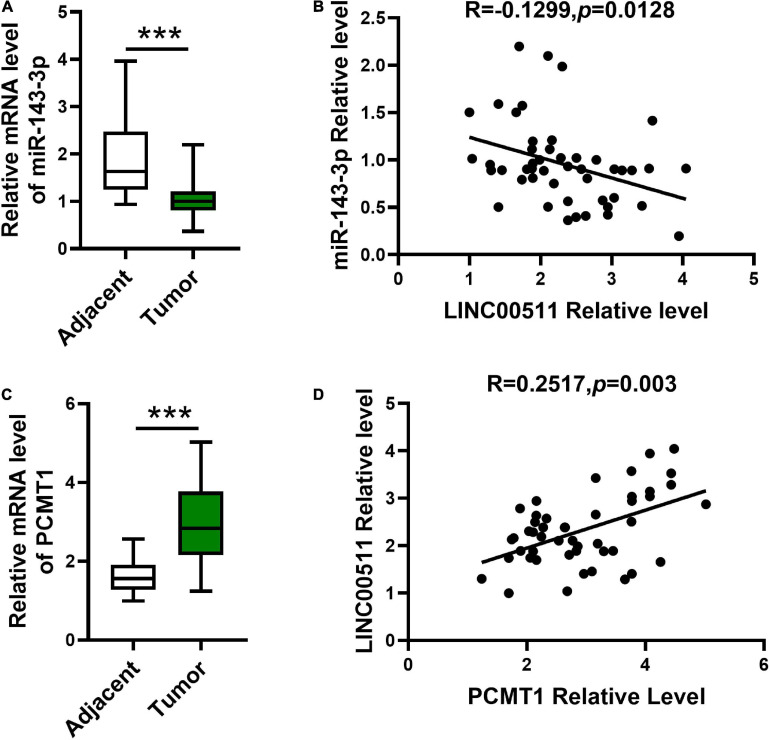
Expression and the correlations between LINC00511 and miR-143-3p and PCMT1 expression. **(A)** The level of miR-143-3p was measured in fresh bladder samples and non-cancer tissues using quantitative RT-PCR (qRT-PCR). *n* = 47; ***p < 0.001. **(B)** Correlations between the LINC00511 and miR-143-3p were analyzed using Spearman rank correlation analysis. *R* = -0.1299, *p* = 0.0128. **(C)** PCMT1 level in fresh bladder cancer samples and non-cancer tissues was analyzed using qRT-PCR. *n* = 47; ***p < 0.001. **(D)** Correlations between the LINC005111 and PCMT1 were analyzed using Spearman rank correlation analysis. *R* = 0.2517, *p* = 0.002.

Next, we explored miR-143-3p and PCMT1 expression in bladder tissues and corresponding adjacent tissues using quantitative RT-PCR. The miR-143-3p was significantly decreased in the bladder tissues compared with the related non-cancerous tissues (Figure 8A). In addition, an opposite correlation was identified between LINC00511 and miR-143-3p expression levels (Figure 8B). PCMT1 was significantly up-regulated in the bladder tissues (Figure 8C). Furthermore, PCMT1 was a positively correlated gene of LINC00511 (Figure 8D).

## Discussion

With the development of next-generation sequencing technology, lncRNA has become a hot spot in the field of non-coding RNA research in recent years. LncRNA can regulate gene expression in various ways and is widely involved in the up-regulation or down-regulation of oncogenes or tumor suppressor genes and plays a very important regulatory function in various physiological and pathological activities such as cell differentiation, proliferation, metabolism, apoptosis, and senescence (Ji et al., 2020; Kanwal et al., 2020). The differential expression of lncRNA in malignant tumors and the molecular mechanism of action have been gradually investigated, and more and more lncRNAs have been found to be closely related to the occurrence and development of tumors. Some lncRNAs, such as ITGB1, UCA1, and DUXAP10, have been reported to play a key role in bladder cancer, significantly affecting the proliferation, migration, invasion, drug resistance, and other malignant biological behaviors of bladder cancer cells, and are correlated with early diagnosis, recurrence, metastasis, and prognosis of bladder cancer (Lv et al., 2018; Avgeris et al., 2019; Dai et al., 2019). LINC00511 is a newly discovered lncRNA with an oncogenic function, which can promote the proliferation and invasion of tumor cells by regulating miRNA expression (Wu et al., 2020; Zhang et al., 2020). In addition, it has been reported that knockdown of LINC00511 promoted apoptosis of bladder cancer cells via suppressing Wnt/β-catenin signaling pathway (Li et al., 2018). The results of our study showed that the expression of LINC00511 in bladder cancer tissues and cell lines was significantly increased, indicating that LINC00511 may act as an oncogene in the occurrence and development of bladder cancer. In this study, MTT and Matrigel invasion assay showed that high expression of LINC00511 significantly increased the proliferation and invasion of bladder cancer cells.

One of the mechanisms of lncRNAs in tumors is the “molecular sponge” effect; that is, through competitive binding of miRNA and down-regulation of miRNA expression, the suppression effect of miRNA on its target causes is dried and interfered. Bioinformation technology prediction showed that LINC00511 could combine with miR-143-3p, and miR-143-3p could combine with PCMT1. MiR-143-3p is significantly low expressed in gastric cancer and lung cancer tissues, negatively regulates the occurrence and development of tumors, promotes the growth and metastasis of tumor cells, and plays an obvious role in tumor suppressor genes (He et al., 2020; Lu et al., 2020). Meanwhile, miR-143-3p has been reported to induce apoptosis in granulosa and rectal cancer cells (Zhong et al., 2020; Shan et al., 2021). Therefore, we investigated the effects of LINC00511/miR-143-3p on the proliferation, invasion, and apoptosis of bladder cancer cells. The results of this study showed that forced expression of miR-143-3p could reverse the proliferation invasion and apoptosis resistance caused by LINC00511.

In recent years, PCMT1 has been gradually considered to be involved in tumor occurrence and development. In lung adenocarcinoma, previous studies found that the expression of PCMT1 was significantly higher in patients with clinically advanced or invasive adenocarcinoma than in patients with clinically early or preinvasive adenocarcinoma (Zhao et al., 2012). It is reported that PCMT1 is a predictive biomarker for poor prognosis in surgically resected lung adenocarcinoma. Studies have shown that breast cancer patients with higher PCMT1 expression have significantly lower survival rates than those with lower PCMT1 expression. PCMT1 expression was high in bladder cancer tissues, and the expression level of PCMT1 was related to the metastasis, staging, and grading of bladder cancer. The higher the expression level of PCMT, the shorter the survival time of patients. The results of this study showed that after LINC00511 inhibited the expression of miR-143-3p, the expression of PCMT1 gene was significantly increased, suggesting that LINC00511 may promote the expression of PCMT1 gene by targeting the expression of miR-143-3p.

However, there are some limitations to our study. It was found in our previous study that PCMT did not affect the proliferation ability of cells (Dong et al., 2018). Therefore, the mechanism of the LINC00511 and miR-143-3p on cell proliferation still needs to be further studied.

To sum up, this study showed that LINC00511 was significantly overexpressed in bladder cancer, and silencing LINC00511 could reduce the proliferation and invasion ability of bladder cancer cells, and its molecular mechanism might be to inhibit the expression of miR-143-3p and promote the expression of PCMT1. LINC00511 may provide a new target for molecular therapy of bladder cancer.

## Data Availability Statement

The original contributions presented in the study are included in the article/supplementary material, further inquiries can be directed to the corresponding author.

## Ethics Statement

The studies involving human participants were reviewed and approved by China Medical University. The patients/participants provided their written informed consent to participate in this study. The animal study was reviewed and approved by China Medical University.

## Author Contributions

L-MD and X-LZ designed the study. M-HM and Y-PL performed the experiments. X-YZ collected and analyzed data. D-WX and Y-LL wrote the manuscript. All authors contributed to the article and approved the submitted version.

## Conflict of Interest

The authors declare that the research was conducted in the absence of any commercial or financial relationships that could be construed as a potential conflict of interest.

## Footnotes

1 http://bioinfo.life.hust.edu.cn/lncRNASNP/#!/

2 http://www.microrna.org/

3 http://www.targetscan.org/vert_72/

## References

[B1] Avgeris M. Tsilimantou A. Levis P. Rampias T. Papadimitriou M. Panoutsopoulou K. (2019). Unraveling UCA1 lncRNA prognostic utility in urothelial bladder cancer. *Carcinogenesis* 40 965–974. 10.1093/carcin/bgz045 30815670

[B2] Burger M. Catto J. Dalbagni G. Grossman H. Herr H. Karakiewicz P. (2013). Epidemiology and risk factors of urothelial bladder cancer. *European urology* 63 234–241. 22877502 10.1016/j.eururo.2012.07.033

[B3] Cambier S. Sylvester R. Collette L. Gontero P. Brausi M. van Andel G. (2016). EORTC nomograms and risk groups for predicting recurrence, progression, and disease-specific and overall survival in non-muscle-invasive stage Ta-T1 urothelial bladder cancer patients treated with 1-3 years of maintenance bacillus calmette-guérin. *Eur. Urol.* 69 60–69. 10.1016/j.eururo.2015.06.045 26210894

[B4] Chang S. Boorjian S. Chou R. Clark P. Daneshmand S. Konety B. (2016). Diagnosis and treatment of non-muscle invasive bladder cancer: AUA/SUO guideline. *J. Urol.* 196 1021–1029. 10.1016/j.juro.2016.06.049 27317986

[B5] Chen Z. Zhou L. Liu L. Hou Y. Xiong M. Yang Y. (2020). Single-cell RNA sequencing highlights the role of inflammatory cancer-associated fibroblasts in bladder urothelial carcinoma. *Nat. Commun.* 11:5077. 33033240 10.1038/s41467-020-18916-5 PMC7545162

[B6] Dai L. Chai C. Shen T. Tian Y. Shang Z. Niu Y. (2019). LncRNA ITGB1 promotes the development of bladder cancer through regulating microRNA-10a expression. *Eur. Rev. Med. Pharmacol. Sci.* 23 6858–6867. 31486485 10.26355/eurrev_201908_18725

[B7] Dong L. Li Y. Xue D. Liu Y. (2018). PCMT1 is an unfavorable predictor and functions as an oncogene in bladder cancer. *IUBMB Life* 70 291–299. 10.1002/iub.1717 29517839

[B8] Fabbri M. Girnita L. Varani G. Calin G. (2019). Decrypting noncoding RNA interactions, structures, and functional networks. *Genome Res.* 29 1377–1388. 10.1101/gr.247239.118 31434680 PMC6724670

[B9] Guo X. Xu Y. Wang Z. Wu Y. Chen J. Wang G. (2018). A Linc1405/Eomes complex promotes cardiac mesoderm specification and cardiogenesis. *Cell Stem Cell* 22 893.e–908.e. 29754779 10.1016/j.stem.2018.04.013

[B10] He W. Zhang D. Li D. Zhu D. Geng Y. Wang Q. (2020). Knockdown of Long Non-coding RNA LINC00200 Inhibits Gastric Cancer Progression by Regulating miR-143-3p/SERPINE1 Axis. *Digest. Dis. Sci. [Online ahead of print]* 10.1007/s10620-020-06691-8 33141390

[B11] Ji E. Kim C. Kim W. Lee E. (2020). Role of long non-coding RNAs in metabolic control. *Biochim. Biophys. Acta Gene Regul. Mech.* 1863:194348. 30594638 10.1016/j.bbagrm.2018.12.006

[B12] Kanwal S. Guo X. Ward C. Volpe G. Qin B. Esteban M. (2020). Role of long Non-coding RNAs in reprogramming to induced pluripotency. *Genom. Proteom. Bioinform.* 18 16–25. 10.1016/j.gpb.2019.06.003 32445708 PMC7393543

[B13] Kunze E. Schauer A. Schmitt M. (1983). Histology and histogenesis of two different types of inverted urothelial papillomas. *Cancer* 51 348–358. 10.1002/1097-0142(19830115)51:2<348::aid-cncr2820510231>3.0.co;2-o 6821821

[B14] Li J. Li Y. Meng F. Fu L. Kong C. (2018). Knockdown of long non-coding RNA linc00511 suppresses proliferation and promotes apoptosis of bladder cancer cells via suppressing Wnt/β-catenin signaling pathway. *Biosci. Rep.* 38:BSR20171701. 30042171 10.1042/BSR20171701 PMC6131201

[B15] Li Y. Shi B. Dong F. Zhu X. Liu B. Liu Y. (2020). LncRNA KCNQ1OT1 facilitates the progression of bladder cancer by targeting MiR-218-5p/HS3ST3B1. *Cancer Gene Ther. [Online ahead of print]* 10.1038/s41417-020-00211-6 32820233

[B16] Lu T. Qiu T. Han B. Wang Y. Sun X. Qin Y. (2020). Circular RNA circCSNK1G3 induces HOXA10 signaling and promotes the growth and metastasis of lung adenocarcinoma cells through hsa-miR-143-3p sponging. *Cell. Oncol. (Dordr.) [Online ahead of print]* 10.1007/s13402-020-00565-x 33118120 PMC12980762

[B17] Lv M. Zhong Z. Huang M. Tian Q. Jiang R. Chen J. (2017). lncRNA H19 regulates epithelial-mesenchymal transition and metastasis of bladder cancer by miR-29b-3p as competing endogenous RNA. *Biochim. Biophys. Acta Mol. Cell Res.* 1864 1887–1899. 10.1016/j.bbamcr.2017.08.001 28779971

[B18] Lv X. Ma L. Chen J. Yu R. Li Y. Yan Z. (2018). Knockdown of DUXAP10 inhibits proliferation and promotes apoptosis in bladder cancer cells via PI3K/Akt/mTOR signaling pathway. *Int. J. Oncol.* 52 288–294. 29115412 10.3892/ijo.2017.4195

[B19] Qi F. Tan B. Ma F. Zhu B. Zhang L. Liu X. (2019). System based on CRISPR Cas13a regulates the expression of LncRNA MALAT1 and affects the malignant phenotype of bladder cancer cells. *Int. J. Biol. Sci.* 15 1630–1636. 10.7150/ijbs.33772 31360106 PMC6643210

[B20] Schmitz S. Grote P. Herrmann B. (2016). Mechanisms of long noncoding RNA function in development and disease. *Cell. Mol. Life Sci.* 73 2491–2509. 10.1007/s00018-016-2174-5 27007508 PMC4894931

[B21] Shan T. Tian Z. Li Q. Jiang Y. Liu F. Sun X. (2021). Long intergenic noncoding RNA 00908 promotes proliferation and inhibits apoptosis of colorectal cancer cells by regulating KLF5 expression. *J. Cell. Physiol.* 236 889–899. 10.1002/jcp.29899 33020901

[B22] Statello L. Guo C. Chen L. Huarte M. (2020). Gene regulation by long non-coding RNAs and its biological functions. *Nat. Rev. Mol. Cell Biol.* 22 96–118. 33353982 10.1038/s41580-020-00315-9 PMC7754182

[B23] Sternberg C. Skoneczna I. Kerst J. Albers P. Fossa S. Agerbaek M. (2015). Immediate versus deferred chemotherapy after radical cystectomy in patients with pT3-pT4 or N+ M0 urothelial carcinoma of the bladder (EORTC 30994): an intergroup, open-label, randomised phase 3 trial. *Lancet Oncol.* 16 76–86. 10.1016/s1470-2045(14)71160-x 25498218

[B24] Sun X. Du P. Yuan W. Du Z. Yu M. Yu X. (2015). Long non-coding RNA HOTAIR regulates cyclin J via inhibition of microRNA-205 expression in bladder cancer. *Cell Death Dis.* 6:e1907. 10.1038/cddis.2015.269 26469956 PMC4632298

[B25] Woła̧cewicz M. Hrynkiewicz R. Grywalska E. Suchojad T. Leksowski T. Roliński J. (2020). Immunotherapy in bladder cancer: current methods and future perspectives. *Cancers* 12:1181. 10.3390/cancers12051181 32392774 PMC7281703

[B26] Wu Y. Li L. Wang Q. Zhang L. He C. Wang X. (2020). LINC00511 promotes lung squamous cell carcinoma proliferation and migration via inhibiting miR-150-5p and activating TADA1. *Transl. Lung Cancer Res.* 9 1138–1148. 10.21037/tlcr-19-701 32953492 PMC7481641

[B27] Yang D. Sun Y. Hu L. Zheng H. Ji P. Pecot C. (2013). Integrated analyses identify a master microRNA regulatory network for the mesenchymal subtype in serous ovarian cancer. *Cancer Cell* 23 186–199. 10.1016/j.ccr.2012.12.020 23410973 PMC3603369

[B28] Yao R. Wang Y. Chen L. (2019). Cellular functions of long noncoding RNAs. *Nat. Cell Biol.* 21 542–551. 31048766 10.1038/s41556-019-0311-8

[B29] Zhang X. Wang Y. Zhao A. Kong F. Jiang L. Wang J. (2020). Long Non-Coding RNA LINC00511 accelerates proliferation and invasion in cervical cancer through targeting miR-324-5p/DRAM1 Axis. *Oncotargets Ther.* 13 10245–10256. 10.2147/ott.s255067 33116605 PMC7567551

[B30] Zhao H. Wang F. Wang J. Xie H. Guo J. Liu C. (2012). Maternal PCMT1 gene polymorphisms and the risk of neural tube defects in a Chinese population of Lvliang high-risk area. *Gene* 505 340–344. 10.1016/j.gene.2012.05.035 22647835

[B31] Zhao Y. Zhao L. Li J. Zhong L. (2019). Silencing of long noncoding RNA RP11-476D10.1 enhances apoptosis and autophagy while inhibiting proliferation of papillary thyroid carcinoma cells via microRNA-138-5p-dependent inhibition of LRRK2. *J. Cell. Physiol.* 234 20980–20991. 10.1002/jcp.28702 31102261

[B32] Zhong Y. Li L. Chen Z. Diao S. He Y. Zhang Z. (2020). MIR143 inhibits steroidogenesis and induces apoptosis repressed by H3K27me3 in granulosa cells. *Front. Cell Dev. Biol.* 8:565261. 10.3389/fcell.2020.565261 33195195 PMC7604341

